# 2^4^,4,8-Trioxa-2^1^-aza-1,3,6(1,2)-tri­benzena-2(2,3)-bicyclo­[3.3.0]octa­na­cyclo­octa­phane

**DOI:** 10.1107/S160053680900720X

**Published:** 2009-03-06

**Authors:** P. R. Seshadri, B. Balakrishnan, K. Ilangovan, S. Purushothaman, R. Raghunathan

**Affiliations:** a PostGraduate and Research Department of Physics, Agurchand Manmull Jain College, Chennai 600 114, India; b Department of Physics, P. T. Lee Chengalvaraya Naicker College of Engineering and Technology, Kancheepuram 631 502, India; c PostGraduate and Research Department of Physics, RKM Vivekananda College, Chennai 600 004, India; d Department of Organic Chemistry, University of Madras, Guindy Campus, Chennai 600 025, India

## Abstract

The crystal structure of the title compound, C_26_H_25_NO_3_, was determined as part of an investigation of host–guest and electron donor–acceptor complexes. The oxazole and the pyrrole rings both adopt envelope conformations. The dihedral angle between the two benzene rings directly linked to the oxazole ring is 49.5 (1)°. The crystal structure is stabilized by a C—H⋯π inter­action.

## Related literature

For biological properties of azomethine ylides, see: Chiacchio *et al.* (2003 ▶). For general background, see: Diederich (1991 ▶); Cram & Cram (1994 ▶); Morrison & Hoger (1996 ▶); Padwa (1984 ▶). For reference bond-length data, see: Allen *et al.* (1987 ▶).For puckering and asymmetry parameters, see: Cremer & Pople (1975 ▶); Nardelli (1983 ▶).
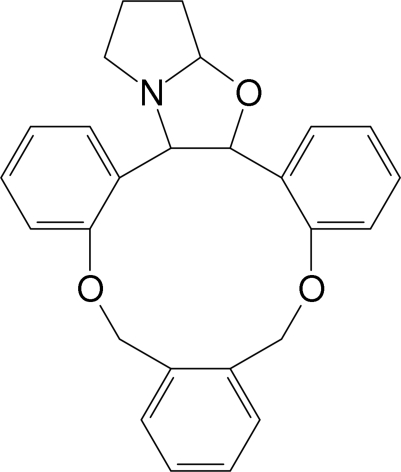


## Experimental

### 

#### Crystal data

C_26_H_25_NO_3_
*M* *_r_* = 399.47
Monoclinic, 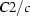

*a* = 31.1942 (7) Å
*b* = 8.3992 (2) Å
*c* = 16.0323 (4) Å
β = 101.468 (1)°
*V* = 4116.70 (17) Å^3^
*Z* = 8
Mo *K*α radiation
μ = 0.08 mm^−1^
*T* = 293 K
0.25 × 0.20 × 0.20 mm

#### Data collection

Bruker Kappa APEXII area-detector diffractometer
Absorption correction: multi-scan (*SADABS*; Sheldrick, 2001 ▶) *T*_min_ = 0.979, *T*_max_ = 0.983
22005 measured reflections
5004 independent reflections
2790 reflections with *I* > 2σ(*I*)
*R*_int_ = 0.029

#### Refinement

*R*[*F* ^2^ > 2σ(*F* ^2^)] = 0.059
*wR*(*F* ^2^) = 0.213
*S* = 1.03
5004 reflections
271 parameters
H-atom parameters constrained
Δρ_max_ = 0.40 e Å^−3^
Δρ_min_ = −0.22 e Å^−3^

### 

Data collection: *APEX2* (Bruker, 2004 ▶); cell refinement: *SAINT* (Bruker, 2004 ▶); data reduction: *SAINT*; program(s) used to solve structure: *SHELXS97* (Sheldrick, 2008 ▶); program(s) used to refine structure: *SHELXL97* (Sheldrick, 2008 ▶); molecular graphics: *ORTEP-3* (Farrugia, 1997 ▶) and *PLATON* (Spek, 2009 ▶); software used to prepare material for publication: *SHELXL97* and *PLATON*.

## Supplementary Material

Crystal structure: contains datablocks I, global. DOI: 10.1107/S160053680900720X/wn2311sup1.cif

Structure factors: contains datablocks I. DOI: 10.1107/S160053680900720X/wn2311Isup2.hkl

Additional supplementary materials:  crystallographic information; 3D view; checkCIF report

## Figures and Tables

**Table 1:** Hydrogen-bond geometry (Å, °)

*D*—H⋯*A*	*D*—H	H⋯*A*	*D*⋯*A*	*D*—H⋯*A*
C7—H7*A*⋯*Cg*1^i^	0.97	2.94	3.827 (3)	153

Symmetry code: (i) 
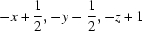
. *Cg*1 is the centroid of the C1–C6 ring.

**Fig. 1.**
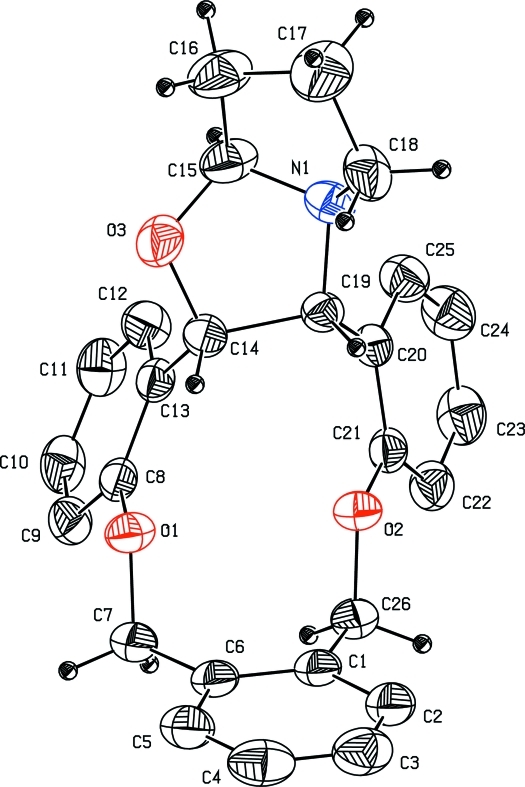
Molecular structure of the title compound, showing 30% probability displacement ellipsoids. Hydrogen atoms are drawn as spheres of arbitrary radius. Hydrogen atoms of the benzene rings are omitted for clarity.

## supplementary crystallographic information

### Comment

The design and synthesis of cyclophanes possessing rigidly defined cavities and shape-persistent structures of molecular dimensions is of interest as molecular hosts in the areas of host-guest and electron donor-acceptor complexes (Diederich, 1991; Cram & Cram, 1994; Morrison & Hoger, 1996). 1,3-Dipolar cycloaddition reactions afford efficient methods for the construction of heterocyclic units in a highly regio- and stereoselective manner (Padwa, 1984). In particular, the chemistry of azomethine ylides has gained significance in recent years as it serves as an expedient route for the construction of nitrogen heterocycles. N and O heterocycles have also been shown to provide useful information about anticancer and antiviral properties (Chiacchio *et al.*, 2003).

In the crystal structure of the title compound, the oxazole ring adopts an envelope conformation with atom C15 displaced by 0.172 (3) Å from the plane of the other ring atoms N1/O3//C14/C19. The puckering parameters (Cremer & Pople, 1975) and asymmetry parameters (Nardelli, 1983) are q_2_ =0.275 (2) Å, φ = 152.3 (5)°, Δ_S_(C15) = 5.7 (2)° and Δ_2_(C19) = 8.5 (2)°. The pyrrole ring also adopts an envelope conformation with atom C18 displaced by 0.208 (3) Å from the plane of the other ring atoms C15/C16/C17/N1. The puckering parameters (Cremer & Pople, 1975) and asymmetry parameters (Nardelli, 1983) are q_2_= 0.329 (3) Å, φ = 144.0 (6)°, Δ_S_(C18) = 0.9 (4)° and Δ_2_(C16) = 16.5 (4)°.

The conformation of the cyclophane ring O1/ C7/ C6/ C1/ C26/ O2/ C21/ C20/ C19/ C14/ C13/ C8 is described by the torsion angles in Table 1. The dihedral angle between the two benzene rings directly linked to the oxazole ring is 49.5 (1)°. The bond lengths (Allen *et al.*, 1987) and bond angles are in agreement with the values reported in literature.

The crystal structure is stabilized by a C—H···π (C7—H7A···Cg1) interaction, where Cg1 is the centroid of the C1—C6 ring.

### Experimental

To a solution of O,O'-coupled salicylaldehyde (bis aldehyde), using *o*-xylylene bromide (2 mmol) in dry acetonitrile (20 ml), was added *L*-proline (1 mmol) under an N_2_ atmosphere. The reaction was refluxed for 4 h. After completion of the reaction, the solvent was distilled off under reduced pressure and the crude product was purified by column chromatography.

### Refinement

All H atoms were positioned geometrically and allowed to ride on their parent atoms, with C—H = 0.93-0.98 Å and *U*_iso_(H) = 1.2*U*_eq_(C).

### Crystal data

**Table:**

C_26_H_25_NO_3_	*F*(000) = 1696
*M**_r_* = 399.47	*D*_x_ = 1.289 Mg m^−^^3^
Monoclinic, *C*2/*c*	Mo *K*α radiation, λ = 0.71073 Å
Hall symbol: C2/c	Cell parameters from 4735 reflections
*a* = 31.1942 (7) Å	θ = 1.3–28.2°
*b* = 8.3992 (2) Å	µ = 0.08 mm^−^^1^
*c* = 16.0323 (4) Å	*T* = 293 K
β = 101.468 (1)°	Block, colourless
*V* = 4116.70 (17) Å^3^	0.25 × 0.20 × 0.20 mm
*Z* = 8	

### Data collection

**Table:**

Bruker Kappa APEXII area-detector diffractometer	5004 independent reflections
Radiation source: fine-focus sealed tube	2790 reflections with *I* > 2σ(*I*)
graphite	*R*_int_ = 0.029
ω scans	θ_max_ = 28.2°, θ_min_ = 1.3°
Absorption correction: multi-scan (*SADABS*; Sheldrick, 2001)	*h* = −40→41
*T*_min_ = 0.979, *T*_max_ = 0.983	*k* = −11→10
22005 measured reflections	*l* = −20→21

### Refinement

**Table:**

Refinement on *F*^2^	Primary atom site location: structure-invariant direct methods
Least-squares matrix: full	Secondary atom site location: difference Fourier map
*R*[*F*^2^ > 2σ(*F*^2^)] = 0.059	Hydrogen site location: inferred from neighbouring sites
*wR*(*F*^2^) = 0.213	H-atom parameters constrained
*S* = 1.03	*w* = 1/[σ^2^(*F*_o_^2^) + (0.1118*P*)^2^ + 1.2039*P*] where *P* = (*F*_o_^2^ + 2*F*_c_^2^)/3
5004 reflections	(Δ/σ)_max_ < 0.001
271 parameters	Δρ_max_ = 0.40 e Å^−^^3^
0 restraints	Δρ_min_ = −0.22 e Å^−^^3^

### Special details

**Table:**

Geometry. All e.s.d.'s (except the e.s.d. in the dihedral angle between two l.s. planes) are estimated using the full covariance matrix. The cell e.s.d.'s are taken into account individually in the estimation of e.s.d.'s in distances, angles and torsion angles; correlations between e.s.d.'s in cell parameters are only used when they are defined by crystal symmetry. An approximate (isotropic) treatment of cell e.s.d.'s is used for estimating e.s.d.'s involving l.s. planes.
Refinement. Refinement of *F*^2^ against ALL reflections. The weighted *R*-factor *wR* and goodness of fit *S* are based on *F*^2^, conventional *R*-factors *R* are based on *F*, with *F* set to zero for negative *F*^2^. The threshold expression of *F*^2^ > σ(*F*^2^) is used only for calculating *R*-factors(gt) *etc*. and is not relevant to the choice of reflections for refinement. *R*-factors based on *F*^2^ are statistically about twice as large as those based on *F*, and *R*- factors based on ALL data will be even larger.

### Fractional atomic coordinates and isotropic or equivalent isotropic displacement parameters (Å^2^)

**Table:**

	*x*	*y*	*z*	*U*_iso_*/*U*_eq_	
O1	0.20831 (5)	1.12120 (17)	0.44961 (9)	0.0625 (4)	
O2	0.14559 (5)	0.87432 (17)	0.35663 (10)	0.0594 (4)	
O3	0.11764 (7)	1.3961 (2)	0.32924 (11)	0.0843 (6)	
N1	0.06599 (6)	1.2386 (2)	0.24650 (12)	0.0645 (5)	
C1	0.21940 (7)	0.7919 (3)	0.37217 (15)	0.0599 (6)	
C2	0.22911 (10)	0.7010 (3)	0.30588 (17)	0.0781 (8)	
H2	0.2090	0.6262	0.2794	0.094*	
C3	0.26785 (11)	0.7196 (4)	0.27879 (19)	0.0905 (9)	
H3	0.2739	0.6577	0.2345	0.109*	
C4	0.29724 (11)	0.8295 (4)	0.3174 (2)	0.0923 (9)	
H4	0.3236	0.8419	0.2993	0.111*	
C5	0.28860 (8)	0.9222 (3)	0.38261 (18)	0.0752 (7)	
H5	0.3090	0.9971	0.4081	0.090*	
C6	0.24927 (8)	0.9047 (3)	0.41087 (15)	0.0607 (6)	
C7	0.24064 (8)	1.0041 (3)	0.48294 (15)	0.0668 (6)	
H7A	0.2302	0.9376	0.5242	0.080*	
H7B	0.2674	1.0560	0.5112	0.080*	
C8	0.17635 (7)	1.1527 (2)	0.49412 (13)	0.0527 (5)	
C9	0.18214 (9)	1.1313 (3)	0.58168 (14)	0.0665 (6)	
H9	0.2089	1.0964	0.6125	0.080*	
C10	0.14848 (10)	1.1616 (3)	0.62241 (16)	0.0745 (7)	
H10	0.1523	1.1456	0.6808	0.089*	
C11	0.10922 (10)	1.2155 (3)	0.57754 (17)	0.0774 (7)	
H11	0.0862	1.2343	0.6052	0.093*	
C12	0.10395 (8)	1.2421 (3)	0.49096 (16)	0.0704 (7)	
H12	0.0774	1.2816	0.4612	0.084*	
C13	0.13715 (7)	1.2113 (2)	0.44787 (13)	0.0525 (5)	
C14	0.13172 (7)	1.2381 (3)	0.35333 (14)	0.0563 (5)	
H14	0.1603	1.2224	0.3381	0.068*	
C15	0.07270 (10)	1.3900 (3)	0.28885 (17)	0.0798 (8)	
H15	0.0538	1.4013	0.3306	0.096*	
C16	0.06289 (13)	1.5177 (4)	0.22115 (19)	0.1011 (10)	
H16A	0.0388	1.5845	0.2301	0.121*	
H16B	0.0884	1.5840	0.2213	0.121*	
C17	0.05085 (14)	1.4273 (4)	0.1393 (2)	0.1170 (12)	
H17A	0.0193	1.4220	0.1209	0.140*	
H17B	0.0631	1.4782	0.0950	0.140*	
C18	0.06941 (10)	1.2667 (3)	0.15755 (16)	0.0816 (8)	
H18A	0.0528	1.1882	0.1201	0.098*	
H18B	0.0997	1.2631	0.1511	0.098*	
C19	0.09799 (7)	1.1284 (2)	0.29399 (13)	0.0525 (5)	
H19	0.1133	1.0770	0.2534	0.063*	
C20	0.07809 (7)	0.9999 (3)	0.33905 (12)	0.0519 (5)	
C21	0.10425 (7)	0.8702 (2)	0.37255 (13)	0.0540 (5)	
C22	0.08812 (9)	0.7507 (3)	0.41656 (16)	0.0687 (7)	
H22	0.1058	0.6657	0.4391	0.082*	
C23	0.04511 (10)	0.7597 (3)	0.42659 (19)	0.0816 (8)	
H23	0.0338	0.6801	0.4563	0.098*	
C24	0.01903 (9)	0.8843 (4)	0.3933 (2)	0.0832 (8)	
H24	−0.0099	0.8886	0.4001	0.100*	
C25	0.03556 (8)	1.0033 (3)	0.34981 (16)	0.0698 (7)	
H25	0.0176	1.0875	0.3273	0.084*	
C26	0.17676 (7)	0.7624 (3)	0.39923 (16)	0.0657 (6)	
H26A	0.1668	0.6548	0.3845	0.079*	
H26B	0.1804	0.7750	0.4604	0.079*	

### Atomic displacement parameters (Å^2^)

**Table:**

	*U*^11^	*U*^22^	*U*^33^	*U*^12^	*U*^13^	*U*^23^
O1	0.0701 (10)	0.0589 (9)	0.0583 (9)	0.0164 (7)	0.0121 (8)	0.0064 (7)
O2	0.0591 (9)	0.0554 (9)	0.0626 (9)	0.0100 (7)	0.0094 (7)	0.0100 (7)
O3	0.1190 (16)	0.0529 (10)	0.0703 (11)	−0.0025 (9)	−0.0067 (10)	0.0091 (8)
N1	0.0603 (12)	0.0722 (13)	0.0579 (11)	0.0056 (9)	0.0046 (9)	0.0161 (9)
C1	0.0632 (14)	0.0525 (12)	0.0595 (13)	0.0157 (10)	0.0015 (11)	0.0063 (10)
C2	0.0860 (19)	0.0707 (16)	0.0692 (16)	0.0215 (13)	−0.0045 (14)	−0.0063 (13)
C3	0.098 (2)	0.100 (2)	0.0741 (18)	0.0352 (18)	0.0192 (17)	−0.0050 (16)
C4	0.083 (2)	0.111 (2)	0.089 (2)	0.0345 (18)	0.0320 (17)	0.0158 (19)
C5	0.0646 (16)	0.0723 (16)	0.0863 (18)	0.0094 (12)	0.0089 (13)	0.0138 (14)
C6	0.0640 (14)	0.0521 (12)	0.0622 (14)	0.0154 (10)	0.0035 (11)	0.0072 (10)
C7	0.0656 (15)	0.0626 (14)	0.0653 (15)	0.0142 (11)	−0.0032 (11)	0.0013 (11)
C8	0.0643 (13)	0.0428 (11)	0.0487 (11)	−0.0024 (9)	0.0059 (10)	−0.0038 (8)
C9	0.0797 (16)	0.0666 (15)	0.0478 (12)	−0.0007 (12)	−0.0003 (11)	−0.0036 (10)
C10	0.104 (2)	0.0747 (16)	0.0452 (12)	−0.0122 (14)	0.0145 (14)	−0.0080 (11)
C11	0.0869 (19)	0.0867 (19)	0.0649 (16)	−0.0075 (14)	0.0302 (15)	−0.0144 (13)
C12	0.0687 (15)	0.0807 (17)	0.0619 (15)	0.0058 (12)	0.0136 (12)	−0.0048 (12)
C13	0.0616 (13)	0.0468 (11)	0.0486 (11)	−0.0020 (9)	0.0097 (10)	−0.0061 (9)
C14	0.0621 (13)	0.0550 (13)	0.0502 (12)	0.0010 (9)	0.0074 (10)	0.0041 (9)
C15	0.104 (2)	0.0718 (17)	0.0639 (15)	0.0302 (14)	0.0183 (15)	0.0094 (12)
C16	0.144 (3)	0.080 (2)	0.0763 (19)	0.0394 (18)	0.0144 (18)	0.0199 (16)
C17	0.178 (4)	0.086 (2)	0.078 (2)	0.009 (2)	0.004 (2)	0.0264 (18)
C18	0.102 (2)	0.0821 (18)	0.0520 (14)	−0.0150 (14)	−0.0052 (13)	0.0114 (12)
C19	0.0564 (12)	0.0537 (12)	0.0461 (11)	0.0062 (9)	0.0074 (9)	0.0007 (9)
C20	0.0541 (13)	0.0550 (12)	0.0445 (11)	0.0000 (9)	0.0046 (9)	−0.0044 (9)
C21	0.0617 (13)	0.0503 (12)	0.0473 (11)	−0.0052 (9)	0.0044 (9)	−0.0047 (9)
C22	0.0781 (17)	0.0597 (14)	0.0647 (15)	−0.0086 (11)	0.0053 (12)	0.0034 (11)
C23	0.092 (2)	0.0769 (18)	0.0759 (18)	−0.0253 (15)	0.0164 (15)	0.0091 (14)
C24	0.0637 (16)	0.094 (2)	0.093 (2)	−0.0116 (14)	0.0185 (14)	0.0062 (16)
C25	0.0621 (15)	0.0729 (16)	0.0733 (16)	−0.0012 (11)	0.0109 (12)	0.0062 (12)
C26	0.0678 (15)	0.0525 (13)	0.0717 (15)	0.0072 (10)	0.0019 (12)	0.0098 (11)

### Geometric parameters (Å, °)

**Table:**

O1—C8	1.362 (2)	C11—H11	0.9300
O1—C7	1.433 (2)	C12—C13	1.379 (3)
O2—C21	1.364 (3)	C12—H12	0.9300
O2—C26	1.425 (2)	C13—C14	1.508 (3)
O3—C15	1.423 (3)	C14—C19	1.569 (3)
O3—C14	1.427 (3)	C14—H14	0.9800
N1—C15	1.437 (3)	C15—C16	1.513 (4)
N1—C19	1.459 (3)	C15—H15	0.9800
N1—C18	1.470 (3)	C16—C17	1.498 (5)
C1—C6	1.386 (3)	C16—H16A	0.9700
C1—C2	1.390 (3)	C16—H16B	0.9700
C1—C26	1.500 (3)	C17—C18	1.475 (4)
C2—C3	1.372 (4)	C17—H17A	0.9700
C2—H2	0.9300	C17—H17B	0.9700
C3—C4	1.360 (5)	C18—H18A	0.9700
C3—H3	0.9300	C18—H18B	0.9700
C4—C5	1.373 (4)	C19—C20	1.501 (3)
C4—H4	0.9300	C19—H19	0.9800
C5—C6	1.398 (3)	C20—C25	1.372 (3)
C5—H5	0.9300	C20—C21	1.402 (3)
C6—C7	1.493 (3)	C21—C22	1.378 (3)
C7—H7A	0.9700	C22—C23	1.385 (4)
C7—H7B	0.9700	C22—H22	0.9300
C8—C13	1.388 (3)	C23—C24	1.367 (4)
C8—C9	1.391 (3)	C23—H23	0.9300
C9—C10	1.366 (4)	C24—C25	1.376 (4)
C9—H9	0.9300	C24—H24	0.9300
C10—C11	1.368 (4)	C25—H25	0.9300
C10—H10	0.9300	C26—H26A	0.9700
C11—C12	1.383 (4)	C26—H26B	0.9700
			
C8—O1—C7	118.15 (17)	O3—C15—N1	106.52 (19)
C21—O2—C26	118.28 (17)	O3—C15—C16	110.0 (3)
C15—O3—C14	108.19 (18)	N1—C15—C16	107.4 (2)
C15—N1—C19	107.11 (18)	O3—C15—H15	110.9
C15—N1—C18	106.54 (19)	N1—C15—H15	110.9
C19—N1—C18	115.7 (2)	C16—C15—H15	110.9
C6—C1—C2	119.2 (2)	C17—C16—C15	104.4 (3)
C6—C1—C26	122.7 (2)	C17—C16—H16A	110.9
C2—C1—C26	118.1 (2)	C15—C16—H16A	110.9
C3—C2—C1	121.3 (3)	C17—C16—H16B	110.9
C3—C2—H2	119.4	C15—C16—H16B	110.9
C1—C2—H2	119.4	H16A—C16—H16B	108.9
C4—C3—C2	119.3 (3)	C18—C17—C16	105.6 (2)
C4—C3—H3	120.3	C18—C17—H17A	110.6
C2—C3—H3	120.3	C16—C17—H17A	110.6
C3—C4—C5	121.0 (3)	C18—C17—H17B	110.6
C3—C4—H4	119.5	C16—C17—H17B	110.6
C5—C4—H4	119.5	H17A—C17—H17B	108.7
C4—C5—C6	120.3 (3)	N1—C18—C17	103.8 (2)
C4—C5—H5	119.8	N1—C18—H18A	111.0
C6—C5—H5	119.8	C17—C18—H18A	111.0
C1—C6—C5	118.8 (2)	N1—C18—H18B	111.0
C1—C6—C7	121.3 (2)	C17—C18—H18B	111.0
C5—C6—C7	119.8 (2)	H18A—C18—H18B	109.0
O1—C7—C6	108.49 (18)	N1—C19—C20	113.70 (17)
O1—C7—H7A	110.0	N1—C19—C14	104.53 (17)
C6—C7—H7A	110.0	C20—C19—C14	114.96 (17)
O1—C7—H7B	110.0	N1—C19—H19	107.8
C6—C7—H7B	110.0	C20—C19—H19	107.8
H7A—C7—H7B	108.4	C14—C19—H19	107.8
O1—C8—C13	116.60 (18)	C25—C20—C21	118.2 (2)
O1—C8—C9	122.8 (2)	C25—C20—C19	123.3 (2)
C13—C8—C9	120.6 (2)	C21—C20—C19	118.58 (18)
C10—C9—C8	120.0 (2)	O2—C21—C22	124.7 (2)
C10—C9—H9	120.0	O2—C21—C20	114.11 (18)
C8—C9—H9	120.0	C22—C21—C20	121.2 (2)
C9—C10—C11	120.2 (2)	C21—C22—C23	118.7 (2)
C9—C10—H10	119.9	C21—C22—H22	120.6
C11—C10—H10	119.9	C23—C22—H22	120.6
C10—C11—C12	119.8 (2)	C24—C23—C22	120.8 (2)
C10—C11—H11	120.1	C24—C23—H23	119.6
C12—C11—H11	120.1	C22—C23—H23	119.6
C13—C12—C11	121.4 (2)	C23—C24—C25	120.0 (3)
C13—C12—H12	119.3	C23—C24—H24	120.0
C11—C12—H12	119.3	C25—C24—H24	120.0
C12—C13—C8	117.9 (2)	C20—C25—C24	121.2 (2)
C12—C13—C14	122.0 (2)	C20—C25—H25	119.4
C8—C13—C14	120.04 (19)	C24—C25—H25	119.4
O3—C14—C13	112.20 (18)	O2—C26—C1	108.19 (18)
O3—C14—C19	104.38 (17)	O2—C26—H26A	110.1
C13—C14—C19	116.72 (18)	C1—C26—H26A	110.1
O3—C14—H14	107.7	O2—C26—H26B	110.1
C13—C14—H14	107.7	C1—C26—H26B	110.1
C19—C14—H14	107.7	H26A—C26—H26B	108.4
			
C6—C1—C2—C3	0.7 (4)	C19—N1—C15—C16	145.9 (2)
C26—C1—C2—C3	−178.8 (2)	C18—N1—C15—C16	21.5 (3)
C1—C2—C3—C4	−0.2 (4)	O3—C15—C16—C17	115.0 (3)
C2—C3—C4—C5	−0.3 (5)	N1—C15—C16—C17	−0.5 (4)
C3—C4—C5—C6	0.3 (4)	C15—C16—C17—C18	−20.5 (4)
C2—C1—C6—C5	−0.7 (3)	C15—N1—C18—C17	−34.3 (3)
C26—C1—C6—C5	178.8 (2)	C19—N1—C18—C17	−153.2 (2)
C2—C1—C6—C7	−179.3 (2)	C16—C17—C18—N1	33.6 (4)
C26—C1—C6—C7	0.2 (3)	C15—N1—C19—C20	111.9 (2)
C4—C5—C6—C1	0.2 (3)	C18—N1—C19—C20	−129.5 (2)
C4—C5—C6—C7	178.9 (2)	C15—N1—C19—C14	−14.3 (2)
C8—O1—C7—C6	138.6 (2)	C18—N1—C19—C14	104.3 (2)
C1—C6—C7—O1	−74.0 (3)	O3—C14—C19—N1	−4.3 (2)
C5—C6—C7—O1	107.5 (2)	C13—C14—C19—N1	120.2 (2)
C7—O1—C8—C13	−154.37 (19)	O3—C14—C19—C20	−129.65 (19)
C7—O1—C8—C9	26.9 (3)	C13—C14—C19—C20	−5.2 (3)
O1—C8—C9—C10	−178.5 (2)	N1—C19—C20—C25	−12.9 (3)
C13—C8—C9—C10	2.8 (3)	C14—C19—C20—C25	107.6 (2)
C8—C9—C10—C11	−1.1 (4)	N1—C19—C20—C21	167.67 (18)
C9—C10—C11—C12	−1.2 (4)	C14—C19—C20—C21	−71.9 (2)
C10—C11—C12—C13	1.8 (4)	C26—O2—C21—C22	−10.6 (3)
C11—C12—C13—C8	−0.1 (3)	C26—O2—C21—C20	170.11 (18)
C11—C12—C13—C14	179.4 (2)	C25—C20—C21—O2	178.11 (19)
O1—C8—C13—C12	179.08 (19)	C19—C20—C21—O2	−2.4 (3)
C9—C8—C13—C12	−2.2 (3)	C25—C20—C21—C22	−1.2 (3)
O1—C8—C13—C14	−0.4 (3)	C19—C20—C21—C22	178.2 (2)
C9—C8—C13—C14	178.3 (2)	O2—C21—C22—C23	−178.6 (2)
C15—O3—C14—C13	−105.7 (2)	C20—C21—C22—C23	0.7 (3)
C15—O3—C14—C19	21.6 (2)	C21—C22—C23—C24	0.2 (4)
C12—C13—C14—O3	54.1 (3)	C22—C23—C24—C25	−0.5 (4)
C8—C13—C14—O3	−126.5 (2)	C21—C20—C25—C24	0.9 (3)
C12—C13—C14—C19	−66.3 (3)	C19—C20—C25—C24	−178.5 (2)
C8—C13—C14—C19	113.2 (2)	C23—C24—C25—C20	−0.1 (4)
C14—O3—C15—N1	−31.6 (3)	C21—O2—C26—C1	−179.37 (17)
C14—O3—C15—C16	−147.6 (2)	C6—C1—C26—O2	84.9 (3)
C19—N1—C15—O3	28.2 (2)	C2—C1—C26—O2	−95.5 (2)
C18—N1—C15—O3	−96.2 (2)		

### Hydrogen-bond geometry (Å, °)

**Table:**

*D*—H···*A*	*D*—H	H···*A*	*D*···*A*	*D*—H···*A*
C7—H7A···Cg1^i^	0.97	2.94	3.827 (3)	153

Symmetry codes: (i) −*x*+1/2, −*y*−1/2, −*z*+1.

## References

[bb1] Allen, F. H., Kennard, O., Watson, D. G., Brammer, L., Orpen, A. G. & Taylor, R. (1987). *J. Chem. Soc. Perkin Trans. 2*, pp. S1–19.

[bb2] Bruker (2004). *APEX2* and *SAINT* Bruker AXS Inc., Madison, Wisconsin, USA.

[bb3] Chiacchio, U., Corsaro, A., Iannazzo, D., Piperno, A., Pistara, V., Resifina, A., Romeo, R., Sindona, G. & Romeo, G. (2003). *Tetrahedron Asymmetry*, **14**, 2717–2723.

[bb4] Cram, D. J. & Cram, D. M. (1994). *Container Molecules and Their Guests* Cambridge: Royal Society of Chemistry.

[bb5] Cremer, D. & Pople, J. A. (1975). *J. Am. Chem. Soc* **97**, 1354–1358.

[bb6] Diederich, F. (1991). *Cyclophanes* Cambridge: Royal Society of Chemistry.

[bb7] Farrugia, L. J. (1997). *J. Appl. Cryst.* **30**, 565.

[bb8] Morrison, D. L. & Hoger, S. (1996). *Chem. Commun.* pp. 2313–2314.

[bb9] Nardelli, M. (1983). *Acta Cryst.* C**39**, 1141–1142.

[bb10] Padwa, A. (1984). Editor. *1-3-Dipolar Cycloaddition Chemistry*, Vols. 1 and 2. New York: Wiley.

[bb11] Sheldrick, G. M. (2001). *SADABS* University of Göttingen, Germany.

[bb12] Sheldrick, G. M. (2008). *Acta Cryst.* A**64**, 112–122. 10.1107/S0108767307043930 18156677

[bb13] Spek, A. L. (2009). *Acta Cryst.* D**65**, 148–155. 10.1107/S090744490804362X PMC2631630 19171970

